# High‐risk human papilloma virus was not detected in a Norwegian cohort of oral squamous cell carcinoma of the mobile tongue

**DOI:** 10.1002/cre2.342

**Published:** 2020-11-03

**Authors:** Tine M. Søland, Inger‐Heidi Bjerkli, Jeanette B. Georgsen, Olaf Schreurs, Peter Jebsen, Helene Laurvik, Dipak Sapkota

**Affiliations:** ^1^ Faculty of Dentistry, Institute of Oral Biology University of Oslo Oslo Norway; ^2^ Department of Pathology, Rikshospitalet Oslo University Hospital Oslo Norway; ^3^ Department of Otorhinolaryngology University Hospital of North Norway Tromsø Norway; ^4^ Department of Medical Biology, Faculty of Health Sciences UiT The Arctic University of Norway Tromsø Norway; ^5^ Department of Pathology Aarhus University Hospital Aarhus Denmark

**Keywords:** cancer of head and neck, cancer of the mouth, human papilloma virus, immunohistochemistry, in situ hybridization, squamous cell carcinoma

## Abstract

**Objectives:**

The presence of and the causative role of high‐risk human papilloma virus (HPV) is a subject of controversy in oral squamous cell carcinoma (OSCC). The disagreement can be related to the misclassification of OSCC as oropharyngeal squamous cell carcinoma and/or lack of standard detection methods. This study aimed to examine the presence of transcriptionally active high‐risk HPV in a homogenous Norwegian cohort of primary and second primary OSCC of the mobile tongue (oral tongue squamous cell carcinoma—OTSCC).

**Methods:**

Tissue microarrays containing formalin‐fixed and paraffin‐embedded cores of 146 OTSCC from the anterior 2/3 of the tongue (n = 128 primary and n = 18 second primary) from a multicentric Norwegian cohort were examined for the presence of high‐risk HPV by DNA‐ and RNA‐in situ hybridization (ISH) assays and p16 immunohistochemistry.

**Results:**

Transcriptionally active HPV (*E6/E7* mRNA) was not identified in any of the OTSCC specimens. In parallel, no tumors were positive for HPV by DNA ISH. Although, 61 (42%) OTSCC demonstrated p16 positivity with varying staining intensity and subcellular localization, only two cases demonstrated strong and uniform p16‐staining (both cytoplasmic and nuclear) in >70% of cancer cells. The absence of transcriptionally active high‐risk HPV in this cohort of OTSCC indicates that high‐risk HPV is an unlikely causative factor in the present material.

## 1 INTRODUCTION

The oral cavity is considered to be a separate anatomical location from the oropharynx. Here, squamous cell carcinoma (SCC) accounts for the majority of malignant tumors (Markopoulos, 2012). The 5‐year overall survival for oral squamous cell carcinoma (OSCC) is about 64% and is closely related to the tumor stage (Zanoni et al., 2019). In a recent systematic review, the mobile tongue is shown to be the most common site of oral cancer among the patients below 45 years of age (Hussein et al., 2017). This is of great concern since patients with oral tongue squamous cell carcinoma (OTSCC) have a significantly more unfavorable prognosis than those at other oral cavity sites (Rusthoven, Ballonoff, Raben, & Chen, 2008).

High‐risk human papilloma virus (HPV) is the primary etiological factor in oropharyngeal squamous cell carcinoma (OPSCC) in the Western world (Vokes, Agrawal, & Seiwert, 2015). Accordingly, high‐risk HPV subtypes have been shown to be present in more than 70% of OPSCC (Fossum, Lie, Jebsen, Sandlie, & Mork, 2017). The HPV positive OPSCC is considered to be a biologically distinct entity and is associated with a higher survival rate as compared to the conventional HPV negative (tobacco‐induced) OPSCC (Ang et al., 2010). In 1983, Sÿrjanen proposed that HPV could be a possible etiological factor for a subgroup of OSCC (Syrjänen, Syrjänen, Lamberg, Pyrhönen, & Nuutinen, 1983). Since then, several studies have focused on HPV detection in OSCC, however, with conflicting results (Lewis Jr et al., 2012; Yete, D'Souza, & Saranath, 2018). Firstly, misclassification of OPSCC as OSCC makes it difficult to analyze the results (Mirghani, Amen, Moreau, & Lacau St Guily, 2015). Secondly, lack of standard methodological approach for HPV testing can significantly lead to over‐ or underestimation of HPV positivity (Braakhuis, Brakenhoff, Meijer, Snijders, & Leemans, 2009; Lewis Jr et al., 2012; Westra, 2014). In a systematic literature review of approximately 4,000 oral cavity cancer specimens, the weighted prevalence of polymerase chain reaction (PCR)‐based HPV DNA detection was found to be 20.2% (Isayeva, Li, D, & Brandwein‐Gensler, 2012). However, the high sensitivity of DNA PCR analysis increases the risk of false positive results. Moreover, HPV DNA detection does not distinguish an active (driver) HPV infection from passenger/bystander infection (Kim, Lewis Jr., & Chen, 2018; Madrigal, Bishop, & Faquin, 2018). In recent years, the mRNA *E6/E7* in situ hybridization (ISH) technique has become increasing popular and it allows direct visualization of viral transcripts in routinely processed tissues, thereby reflecting the active HPV infection (Wang et al., 2014). To our knowledge, only a few studies using relatively limited numbers of OSCC have evaluated the presence of high‐risk HPV in OSCC by this technique (Bishop et al., 2012; Lewis Jr et al., 2012; Poling et al., 2014). Their results indicate that high‐risk HPV prevalence is very low in OSCC and challenge the view that HPV is a possible etiological factor in OSCC. This underscores the importance of studies aimed at identifying active HPV infection in a large and homogenous OSCC cohort.

The current work represents a sub‐study of a joint initiative (Norwegian Oral Cancer [NOROC] multicenter study) between the four University hospitals in Norway treating OSCC (Bjerkli et al., 2020). Here, 146 OTSCC diagnosed in Norway from 2005 until 2010 were included. The hypothesis of the current study was that high‐risk HPV infection is uncommon in OTSCC in the Norwegian population. Here, we compared three different and independent approaches for high‐risk HPV detection (*E6/E7* mRNA and DNA ISH and p16 IHC) in OTSCC.

## 2 MATERIAL AND METHODS

### 2.1 OTSCC selection and extraction of clinical data

**FIGURE 1:**
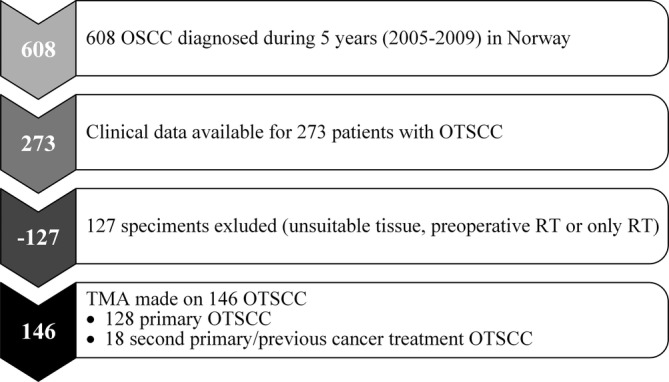
Flowchart illustrating the selection procedure for OTSCC used in this study. OSCC, oral squamous cell carcinoma; OTSCC, oral tongue squamous cell carcinoma; RT, radiation therapy; TMA, tissue microarray

The study was approved by the Northern Norwegian Regional Committee for Medical Research Ethics (REK Nord; 2013/1786 and 2015/1381). In this multicenter study, all conventional OSCC cases diagnosed between January 1, 2005 and December 31, 2009 at the four Norwegian university hospitals treating head and neck cancer (Oslo, Bergen, Trondheim, and Tromsø) were retrospectively identified. Using ICD‐10 codes (C02‐C06) except for codes C05.1 and C05.2 (oropharyngeal sites, and cancer of the external upper or lower lip/vermilion), a total of 608 OSCC patients were identified from the electronic health record. Two hundred and seventy‐three OTSCC with clinical data were identified. Of the 273 OTSCC, 146 (128 primary + 18 second primary) were included in this study (for details of the exclusion criteria, see Figure 1). Unidentifiable clinical data were recorded in a web‐based case report form (CRF). relevant patient data, ICD‐10 diagnosis, TNM classification, treatment received, and minimum of 5 years follow‐up (last follow‐up date June 1, 2015) were registered. The patients were classified according to TNM 5th Edition 2005 UICC (Wittekind, Greene, Hutter, Klimpfinger, & Sobin, 2005).

### 2.2 Tissue microarray generation

Tissue microarrays were constructed from formalin‐fixed, paraffin‐embedded (FFPE) tissue blocks in a fully automated tissue microarray machine (TMA Master II, 3DHISTECH). Two to four tissue cores (both invasive front and more superficial parts of the tumors) with a diameter of 2 mm were arrayed on the recipient paraffin blocks. The stained TMA‐sections were scanned (Pannoramic® MIDI scanner, Thermo Fisher Scientific) and evaluated using the CaseViewer™ software (3dhistech.com). For scanned images with inadequate focus, the original glass slides were examined by a Leitz Aristoplan microscope.

### 2.3 p16 immunohistochemistry and scoring

Immunohistochemistry (IHC) was performed on TMAs on a Ventana Benchmark Ultra automated immunostainer (Ventana Medical Systems, VMS, Tucson, Arizona), using a mouse monoclonal antibody clone E6H4 (CINtec® p16 Histology, VMS #805‐4713). Bound antibody was detected by the biotin‐free ultraView Universal DAB Detection Kit (VMS #760‐500). A known p16‐expressing OPSCC was used as a positive control. Sections incubated with phosphate buffer saline (instead of primary antibody) and with isotype‐matched control (Mouse IgG2a, Sigma‐Aldrich, St. Louis, Missouri, # M9144) were used as negative controls. For details, see Appendix S1. Blinded for the clinicopathological data, the p16‐stained TMA‐sections were scored by TMS, DS, PJ, and HL. p16 expression was evaluated as follows: Score 0: no expression, Score 1: positive staining in <70% of the tumor cells, Score 2: positive staining, either nuclear or cytoplasmic in >70% of the tumor cells, Score 3: Strong and uniform p16‐staining (both cytoplasmic and nuclear) in >70% of cancer cells (Danish‐Head‐and‐Neck‐Cancer‐Group, 2012).

### 2.4 HPV DNA ISH and scoring

Automated in situ hybridizations were carried out on TMAs on a Discovery XT (VMS) instrument using Research ISH UltraMap XT procedure and Ventana products (INFORM HPV III Family 16 Probe (B), #800‐4295). For details, see Appendix S2. Each TMA slide contained HeLa cells as positive staining controls. Additionally, sections of a known HPV positive OPSCC were also used as positive controls. A no probe control containing only RiboHybe and RiboWash served as a negative control.

Blinded for the clinicopathological data, the HPV DNA ISH results were scored by TMS and DS. The in situ results were interpreted following the manufacturer's guidelines (Interpretation Guide for Ventana INFORM HPV Probes ISH). Any blue nuclear dots in the tumor cells were regarded as positive staining and all of the samples were classified binary as either positive or negative.

### 2.5 HPV RNA ISH

Automated RNA in situ hybridizations were carried out on a Discovery Ultra (Ventana Medical Systems, Tucson, Arizona) using the fully automated RNAscope VS HRP assay (#323200 Advanced Cell Diagnostics Inc, Hayward, California). Standard protocols were used for the deparaffinization followed by heat pretreatement using Discovery CC1 and mRNA sample prep protease treatment. For details, see Appendix S3. The RNA quality was controlled in some randomly selected specimens using a probe for the common housekeeping gene PPIB (#313909 ACD). Background signal was investigated with a negative control probe for the bacterial gene DapB (#312039 ACD). Both probes were incubated on full FFPE sections and evaluated according to the manufactures instructions. Sections of FFPE pellets of HeLa cells were used as positive controls.

Blinded for the clinicopathological data, the HPV RNA ISH results were scored by TMS and DS. A positive HPV test result was defined as punctate staining that localized in the cytoplasm and/or nucleus of malignant cells. The RNA ISH staining was scored according to advanced CELL diagnostics guidelines, as described in the Appendix S4.

### 2.6 Statistical analysis

Descriptive statistics (range, mean, median, and frequencies), where applicable, were calculated for continuous and categorical variables using the Statistical Package for the Social Sciences (spss) 26.0 for Windows (SPSS, Inc., Chicago, Illinois).

## 3 RESULTS

This study adheres to the REMARK criteria (McShane et al., 2005). Out of 146 OTSCC, cases with missing tissue cores or containing few/no malignant cells in the array were excluded from the analysis. Following these criteria, two primary cases were excluded from the analysis of p16 and DNA ISH, and three primary cases were omitted from the analysis of the RNA ISH.

The clinicopathological variables for 128 primary OTSCC are summarized in Table 1. In brief, primary OTSCC occurred in 77 males (60.2%) and in 51 females (39.8%). At the time of diagnosis, the median age for the cohort was 65.5 years (range: 25–90 years). The second primary OTSCC occurred in 10 males (55.6%) and 8 females (44.4%). The median age was 72.0 years (range: 42–91 years). Fifteen tumors (83.3%) were cT1/cT2, two were cT3/cT4 (11.1%) while T‐classification was missing for one case.

**TABLE 1:** Clinicopathological variables in 128 patients with primary oral tongue squamous cell carcinoma

*Gender*	*Patients (n = 128) (%)*
Male	77 (60.2)
Female	51 (39.8)
*Age at diagnosis*, *years*	
0–59	43 (33.6)
≥60	85 (66.4)
*Smoking history*	
Never smoker	30 (23.4)
Former smoker	28 (21.9)
Current smoker	56 (43.8)
Unknown	14 (10.9)
*Alcohol consumption*	
Never drinker	12 (9.4)
Seldom (≤1 times weekly)	26 (20.3)
Moderately/heavy drinking (>1 times weekly or daily)	39 (30.5)
Unknown	51 (39.9)
*Tumor differentiation*	
Well	23 (17.9)
Moderately	88 (68.8)
Poor	12 (9.4)
Unknown	5 (3.9)
*cT status*	
cT1/cT2	101 (78.9)
cT3/cT4	25 (19.5)
Unknown	2 (1.6)
*cN status*	
N0	93 (72.7)
N+	31 (24.3)
Unknown	4 (3.2)

### 3.1 p16 immunohistochemistry

**FIGURE 2:**
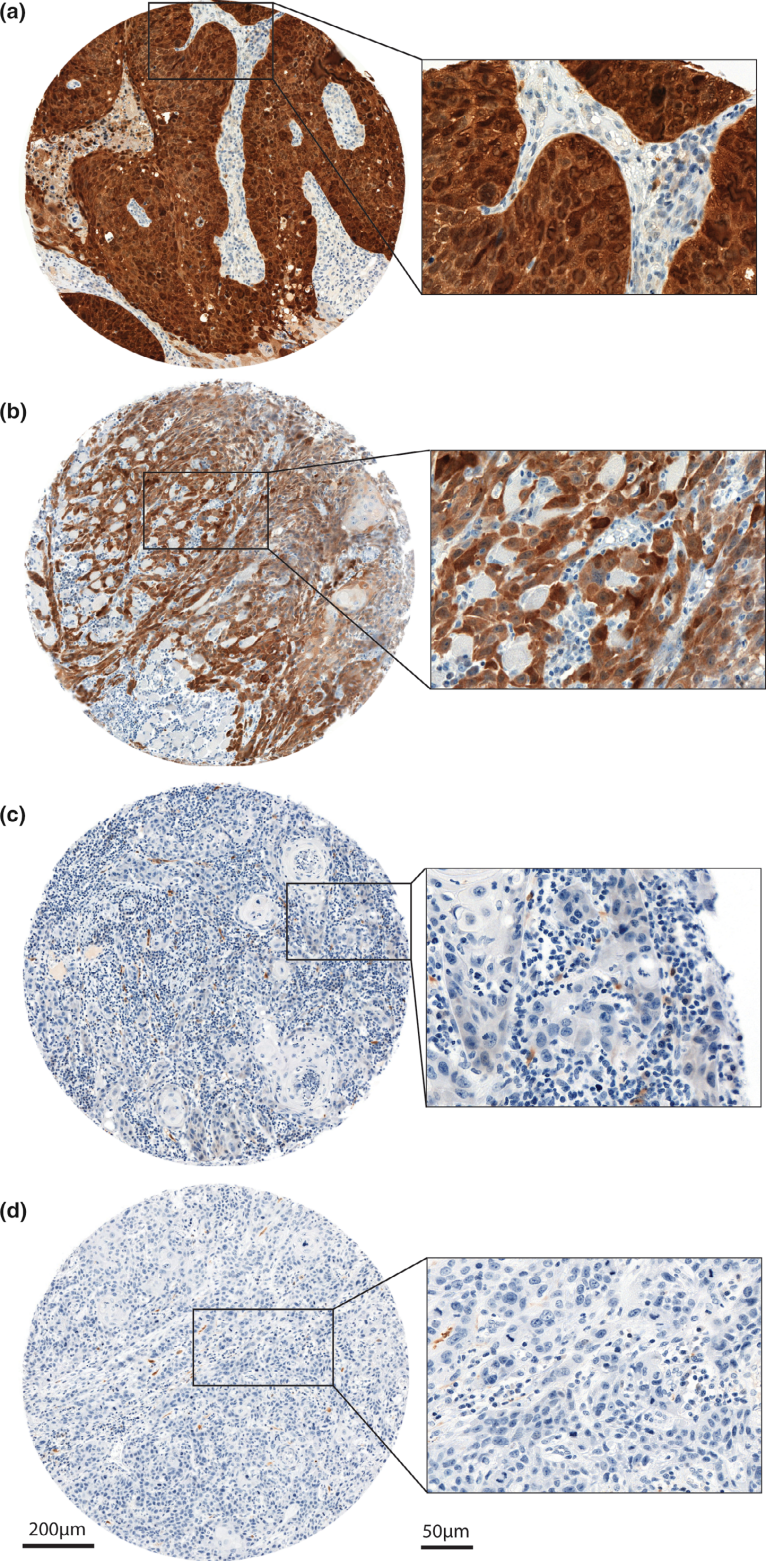
p16 immunohistochemistry in OTSCC. 2A = Strong and uniform p16‐staining (both cytoplasmic and nuclear) in >70% of OTSCC cells, score 3. 2B = cytoplasmic p16 staining in >70% of the OTSCC cells, score 2. 2C = weak cytoplasmic staining in OTSCC cells, score 1. 2D = p16 negative OTSCC cells, score 0

Sixty‐one (42%) OTSCC showed p16 positivity with varying staining intensity and subcellular localization (Figure 2). Among the positives, only two (1%) OTSCC (both primary) demonstrated strong and uniform p16 staining, fulfilling the criteria for Score 3 (Figure 2a). For details on distribution of p16 staining in OTSCC, see Table 2.

**TABLE 2:** Results of p16 immunohistochemical staining of 144 OTSCC

p16 IHC staining	Number (%)	Primary OTSCC number	Second primary OTSCC number
Score 0: No expression	83 (58)	72	11
*Score 1*: *Positive staining in <70% of the tumor cells*	*47 (33)*	*42*	*5*
Score 2: Positive staining, either nuclear or cytoplasmic in >70% of the tumor cells	12 (8)	10	2
*Score 3*: *Strong and uniform p16‐staining (both cytoplasmic and nuclear) in >70% of cancer cells*	*2 (1)*	*2*	*0*

### 3.2 HPV DNA ISH

**FIGURE 3:**
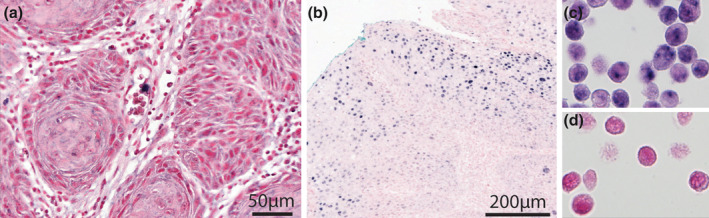
3A = HPV DNA negative OTSCC. 3B = HPV DNA positive OPSCC (positive control). 3C = HPV DNA positive HeLa cells (positive control). 3D = negative no probe control (in situ without probe) in HeLa cells. OPSCC, oropharyngeal squamous cell carcinoma

Based on the evaluation criteria described above, all tumors tested negative for HPV DNA (Figure 3a), including the two cases with p16 Score 3. The positive controls, a known HPV positive OPSCC (Figure 3b) and HeLa cells (Figure 3c) showed positive staining. The no probe control was negative (Figure 3d).

### 3.3 HPV RNA ISH

**FIGURE 4:**
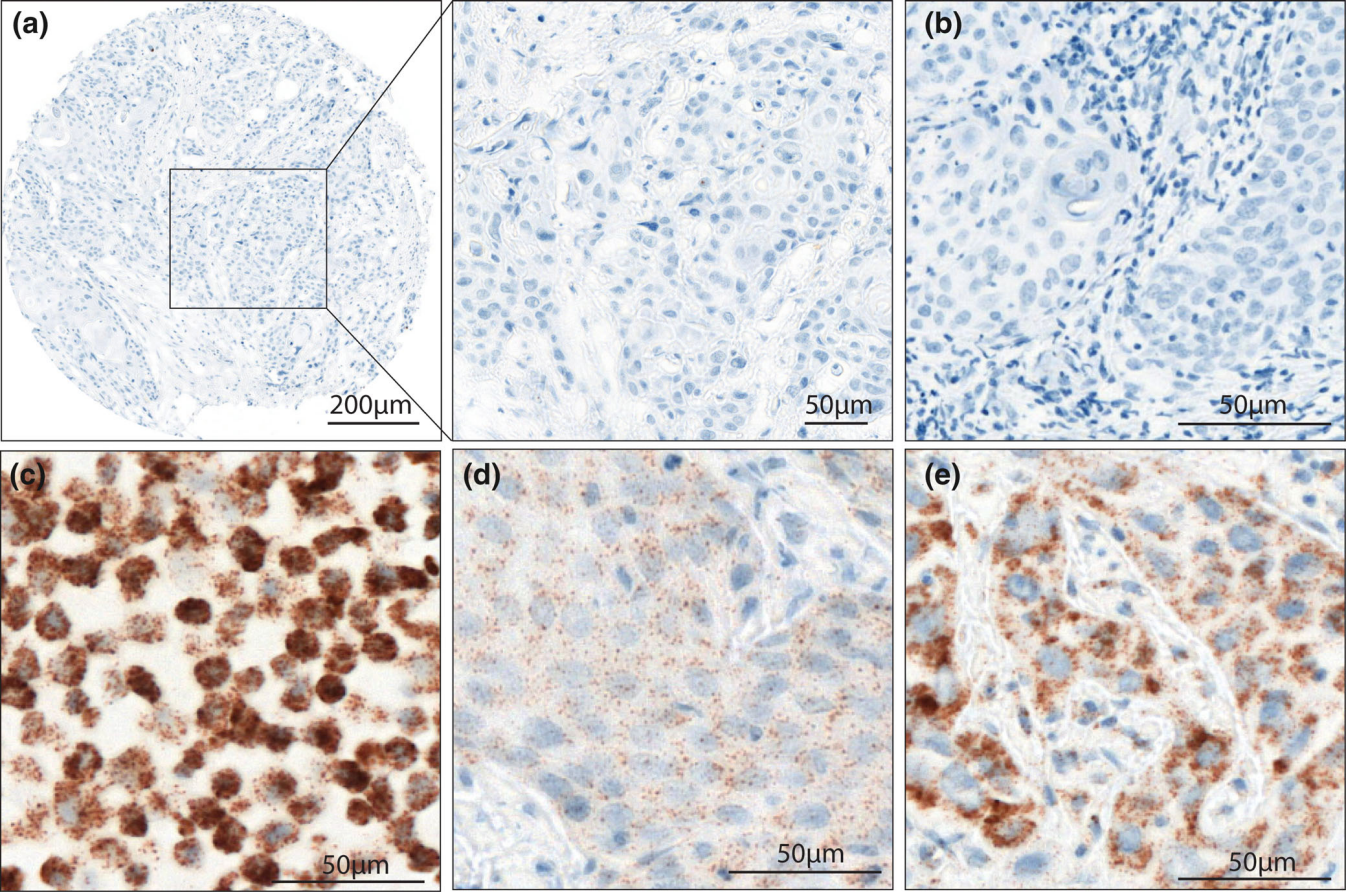
Representative figures for high‐risk HPV RNA in situ hybridization in oral tongue squamous cell carcinoma (OTSCC). 4A = No staining or less than one dot to every 10 cells in a HPV mRNA negative OTSCC. 4B = The bacterial gene dapB was used as negative control. 4C = HPV mRNA positive HeLa cells. 4D and E = Two different OTSCC with PPIB probes were positive (RNA control)

No staining or less than one dot to every 10 cells was observed in all of the OTSCC investigated (Figure 4a). The bacterial gene dapB was used as negative control (Figure 4b). The positive control, HeLa cells, was positive (Figure 4c) and the RNA controls with PPIB probes were positive in two OTSCC (Figure 4d,e).

## 4 DISCUSSION

The pathogenic role of HPV in OPSCC has been well established however, its role in OSCC carcinogenesis is a subject of a controversy. Although several tests are available for HPV detection, there is no consensus on the test method(s) for routine diagnostics of HPV‐related oropharyngeal squamous cell carcinomas/head and neck carcinomas (Kim et al., 2018). Different methods have different detection targets including HPV DNA, RNA, viral oncoproteins like E6/E7, cellular proteins (e.g., p16 protein), or HPV‐specific serum antibodies. Careful selection of the detection technique and viral target is extremely important to obtain reliable and clinically meaningful data. However, the commonly used assays such as p16 IHC and PCR have limitations. For example, p16 overexpression may be caused by molecular mechanisms independent of the presence of high‐risk HPV. DNA or RNA extraction procedures in PCR techniques destroy the tumor tissue context of importance for morphological correlation (Wang et al., 2014). Furthermore, the detection of HPV DNA (either PCR‐based or by ISH) cannot discern an incidental virus from a persistent viral oncogene expression (Westra, 2014). In contrast, RNA ISH probes complementary to *E6/E7* mRNA permit direct visualization of viral transcripts in routinely processed tissues (Ang et al., 2010; Wang et al., 2014). Unfortunately, only a handful of studies have used this approach to examine the HPV infection in OSCC. The current study consisted of a homogenous and a relatively large number of OTSCC specimens (both primary and second primary). Use of three different test methods enabled us to examine the presence of HPV DNA, its transcriptional active form (E6/E7 mRNA) and the HPV‐surrogate marker, p16.

All of the OTSCC specimens in the current Norwegian cohort were negative for *E6/E7* mRNA and HPV DNA. This is in line with the observations reported by Lewis et al., where all of the 45 OSCC examined were negative for HPV E6*/E7* RNA (Lewis Jr et al., 2012). Similarly, only one of 107 OSCC contained transcriptionally active HPV in the study by Bishop et al. (2012). One can suspect that the negative results with RNA/DNA ISH could be related to overfixation of specimens. However, it has been suggested that the excessive cross‐linking related to overfixation could be partially reversed by protease enzyme digestion, as done in the current study. Traditionally, DNA/RNA ISH are considered to have a lower sensitivity, especially in cases with low abundance of the target nucleic acids, as compared to the PCR‐based methods. However, a recent study has demonstrated a better sensitivity and specificity of ISH techniques as compared to PCR in formalin‐fixed paraffin‐embedded OPSCC specimens (Randén‐Brady et al., 2019). Interestingly, the specimens used in that study were collected in the same period of time as in the current study.

Due to the absence of HPV E6/E7 RNA positive carcinomas, we could not characterize their biological or clinical significance. The present HPV DNA results are in agreement with Jaber, Fatani, and Aldhahri (2019). Lewis et al. identified only one HPV DNA positive OSCC out of 45 in their study (Lewis Jr et al., 2012). In contrast, a recent systematic review and meta‐analysis reported a higher prevalence of HPV DNA positivity in South and Central America and Asia, as compared to that in North America and Europe (Ndiaye et al., 2014).

In line with the DNA/RNA ISH results, only two OTSCC were p16 positive (Score 3). However, both of the p16 positive OTSCC were negative for HPV DNA and RNA ISH. This suggests that the p16 expression in those cases might be related to non‐HPV mechanisms and supports the view that p16 is not a reliable surrogate marker for HPV in OTSCC (Bishop et al., 2012; Muller, 2017). A recent meta‐analysis showed that 6.7% of p16 positive head and neck squamous cell carcinomas were not related to transcriptionally active HPV infection and that p16 IHC as a single test does not fulfill the criterion to distinguish between bystander HPV and truly HPV‐driven cancers (Albers, Qian, Kaufmann, & Coordes, 2017).

Although we aimed to investigate conventional OSCC, two non‐keratinizating carcinomas were also included in the current material. One of the two OTSCC with p16 Score 3 staining was a basaloid carcinoma (non‐keratinizating). This is an interesting observation since p16 positive OPSCC usually are non‐keratinizing. However, another non‐keratinizing carcinoma included in this study was p16 negative.

A general increase in the incidence of OTSCC has been reported globally with a shifting trend toward female and/or younger patients with OTSCC (Ng, Iyer, Tan, & Edgren, 2017). In this study, such a trend was not obvious in Norway in the period 2005–2010. Here, the majority of the patients were males and 66% of the patients were 60 years or older. The current study benefitted from the use of a homogenous cohort of OSCC only including carcinomas from the anterior 2/3rd of the tongue. As the Norwegian population is homogeneous regarding ethnic origin, lifestyle, and OSCC‐related risk habits, the carcinomas can be considered similar with respect to etiology and biology, thereby minimizing the potential biases.

Additionally, to minimize the possible bias caused by tumor heterogeneity, tissue cores representing both the invasive front and the more superficial parts of each tumor were included in the TMA block. From the majority of the OTSCC, four tissue cores were prepared. From the rest of the tumors, two tissue cores were made. Four cores should achieve a high degree of concordance when comparing results from whole sections with those of TMA cores (Goethals et al., 2006). A high concordance using triplicate TMA cores (Gulmann & O'Grady, 2003) and even when including only two cores is reported (Camp, Charette, & Rimm, 2000).

## 5 CONCLUSION

None of the 146 OTSCC (128 primary and 18 second primary) diagnosed from 2005 until 2010 were found to be positive for high‐risk HPV. In parallel, only two OTSCC were p16 (Score 3) positive. Our results suggest that high‐risk HPV is an unlikely causative factor in the present material and will not influence future biomarker studies utilizing the current material.

## CONFLICT OF INTEREST

None declared.

## Supporting information


**Appendix** S1: Supporting Information
