# Supplementary material for: High‐risk human papilloma virus was not detected in a Norwegian cohort of oral squamous cell carcinoma of the mobile tongue
Source: Clin Exp Dent Res. 2020 Nov 3;7(1):70–7. doi: 10.1002/cre2.342 (PMC7853882; doi:10.1002/cre2.342)
Supplement: Supplementary file 1 — Appendix S1: Supporting Information [file CRE2-7-70-s001.docx]

### Appendix A. Supplementary data

S1: p16 IHC

For the detection of p16 protein in TMA sections, IHC was performed on a Ventana Benchmark Ultra automated immunostainer (Ventana Medical Systems, VMS, Tucson AZ, USA), using a mouse monoclonal antibody clone E6H4 (CINtec® p16 Histology, VMS #805-4713) and the biotin-free ultraView Universal DAB Detection Kit (VMS #760-500). Briefly, the tissue sections were deparaffinised followed by heat epitope retrieval with Cell Conditioning 1 for 36 minutes at 96 ˚C (mild CC1, VMS #950-124) and quenching of endogenous peroxidase with 3 % hydrogen peroxide for 4 minutes. The slides where incubated with the p16 antibody or an isotype-matched control (Mouse IgG2a, Sigma-Aldrich, St. Louis, MO, # M9144) at the same concentration of 1 µg/ml for 16 minutes at 36 ˚C. Bound antibody was detected by an HRP-multimer labeled secondary antibody cocktail recognizing mouse and rabbit immunoglobulins for 8 min and visualized with 3,3’-diaminobenzidine tetrahydrochloride for 8 minutes before enhancement with copper sulfate for 4 minutes. A known p16- expressing head and neck squamous cell carcinoma was used as positive control. In addition to the isotype-matched control, sections incubated with phosphate buffer saline (instead of primary antibody) was included as a negative control.

S2: DNA *in situ* hybridization for HPV

Automated *in situ* hybridisations were performed on a Discovery XT (VMS) using the following Research ISH UltraMap XT procedure and Ventana products. Sections (4µm) were deparaffinised, followed by heat treatment for 16 minutes at 95 °C using a citrate-based acidic buffer (RiboCC, #760-107) and a protease treatment (Protease 3, #760-2020) for 4 minutes at 37 °C. 200µL Dinitrophenol- (DNP-) labelled probe (INFORM HPV III Family 16 Probe (B), #800-4295) that captures HPV genotypes 16, 18, 31, 33, 35, 45, 52, 56, 58 and 66 was diluted with 75 µL RiboHybe hybridisation buffer (#760-104) and 25 µL RiboWash (#760-105) and a total of 300 µL was added manually on each slide. Denaturation of the probe was performed for 8 minutes at 95 °C followed by a 2 hrs long hybridisation at 52 °C. After hybridisation, three stringency washes ensued with 2 x SSC (RiboWash, #760-105) for 8 min each, at hybridisation temperature. Sections were blocked for 4 minutes with Discovery Antibody Block (#760-4204) before bound probe was detected using a rabbit antibody detecting DNP (#780-4335) for 20 min, followed by an alkaline phosphatase-conjugated anti-rabbit antibody (UltraMap anti-Rb AP, #760-4314) for 16 minutes. Chromogenic signal detection was done by BCIP/NBT for 1 hr (ChromoMap Blue Kit, #760-161). Slides were counterstained manually with 0.1 % Nuclear Fast Red (Gurr, London) in a 5 % aqueous Aluminium sulphate (Sigma-Aldrich) solution for 2 min, washed, dehydrated and coverslips were applied using a xylene-based mounting medium (HistoKit, Assistant).

Each TMA slide contained HeLa cells as positive staining control. Additionally, sections of a known HPV positive OPSCC were also used as positive controls. A no probe control containing RiboHybe and RiboWash only served as a negative control.

S3: RNA *in situ* hybridization for HPV

HPV *E6/E7* mRNA was examined using 5 µm FFPE TMA along with sections of FFPE pellets of human HeLa cells as positive control. The automated RNA *in situ* hybridisations were carried out on a Discovery Ultra (Ventana Medical Systems, Tucson, AZ, USA) using fully automated RNAscope VS HRP assay (#323200 Advanced Cell Diagnostics Inc, Hayward, CA, USA). Standard procedures were used for the deparaffinization followed by heat pretreatement at 100 °C for 32 minutes using Discovery CC1 and mRNA sample prep protease treatment at 37 °C for 16 minutes. Endogenous peroxidase was blocked with DAB inhibitor (#760-224 Ventana medical systems) for 4 minutes. The FFPE TMA slides were incubated with the HPV HR18 cocktail probe for detection of the HPV genotypes: 16, 18, 26, 31, 33, 35, 39, 45, 51, 52, 53, 56, 58, 59, 66, 68, 73, & 82 (#312599 ACD) and hybridized at 43 °C for 2 hours. After the hybridization, the signals were amplified with AMP 5 for 1 hour and detected with the mRNA DAB kit (#760-224 Ventana medical systems). The sections were counterstained with Mayers Hematoxylin for 8 minutes.

To assess the RNA quality in the tissue prior to the HPV *E6/E7* mRNA assay we used a positive probe for the common housekeeping gene PPIB (#313909 ACD) and to assess for background signal we used a negative control probe for the bacterial gene DapB (#312039 ACD). Both probes were incubated on FFPE full sections and evaluated according to the manufactures instructions.

S4: Scoring guideline HPV RNA ISH

Semi-quantitative scoring guideline utilizing the estimated number of punctate dots present within each cell boundary (Advanced Cell Diagnostics 2018).

The staining was categorized according to the following table:

Score 0: No staining or less than 1 dot for every 10 cells (40X magnification)

Score 1: 1–3 dots/cell (visible at 20–40X magnification)

Score 2: 4–10 dots/cell. No or very few dot clusters (visible at 20–40X magnification)

Score 3: <10 dots/cell. Less than 10% positive cells have dot clusters (visible at 20X magnification)

Score 4: >10 dots/cell. More than 10% positive cells have dot clusters (visible at 20X magnification)
